# Recent Developments of Supramolecular Metal-based Structures for Applications in Cancer Therapy and Imaging

**DOI:** 10.7150/thno.31828

**Published:** 2019-05-18

**Authors:** Alexander Pöthig, Angela Casini

**Affiliations:** 1 Department of Chemistry & Catalysis Research Center, Technical University of Munich, Lichtenbergstr. 4, 85747 Garching, Germany.; 2 School of Chemistry, Cardiff University, Main Building, Park Place, Cardiff CF10 3AT, United Kingdom.; 3 Institute for Advanced Study, Technical University of Munich, Department of Chemistry, Lichtenbergstr. 2 a, 85747 Garching, Germany.

**Keywords:** supramolecular metal-based complexes, metallacages, cancer, drug delivery, theranostics.

## Abstract

The biomedical application of discrete supramolecular metal-based structures, including supramolecular coordination complexes (SCCs), is still an emergent field of study. However, pioneering studies over the last 10 years demonstrated the potential of these supramolecular compounds as novel anticancer drugs, endowed with different mechanisms of action compared to classical small-molecules, often related to their peculiar molecular recognition properties. In addition, the robustness and modular composition of supramolecular metal-based structures allows for an incorporation of different functionalities in the same system to enable imaging in cells *via* different modalities, but also active tumor targeting and stimuli-responsiveness. Although most of the studies reported so far exploit these systems for therapy, supramolecular metal-based structures may also constitute ideal scaffolds to develop multimodal *theranostic* agents. Of note, the host-guest chemistry of 3D self-assembled supramolecular structures - within the metallacages family - can also be exploited to design novel drug delivery systems for anticancer chemotherapeutics. In this review, we aim at summarizing the pivotal concepts in this fascinating research area, starting with the main design principles and illustrating representative examples while providing a critical discussion of the state-of-the-art. A section is also included on supramolecular organometallic complexes (SOCs) whereby the (organic) linker is forming the organometallic bond to the metal node, whose biological applications are still to be explored. Certainly, the myriad of possible supramolecular metal-based structures and their almost limitless modularity and tunability suggests that the biomedical applications of such complex chemical entities will continue along this already promising path.

## 1. Introduction

**Figure 1:**
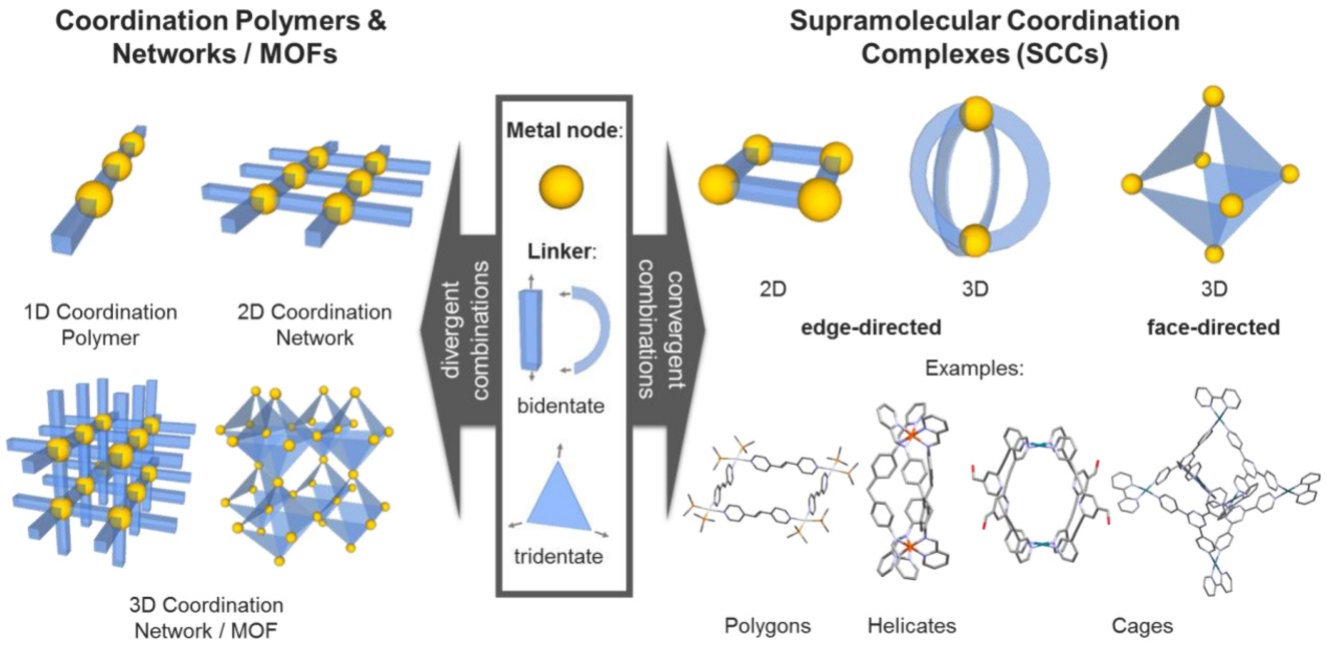
Different types of metal-based assemblies (examples): Coordination polymers and networks formed by divergent (left) and discreet supramolecular complexes formed by convergent combinations of metal nodes and organic linkers (right).

Inspired by the integrative self-sorting observed in nature, a variety of artificial metal-based supramolecular architectures have been developed in the last decades. Hereby, additionally to the regulated and controlled assembly of multi-component (bio) molecules, the metal-containing systems can be designed to include further functionalities in a very diverse way. Most of these supramolecular scaffolds consist of metal ions (Lewis acids) and multidentate ligands (Lewis bases) comprising heteroatoms, i.e. such assemblies are based on “classical” also called “Werner-type” coordination chemistry. Both the coordination geometry of the metal ions or clusters (nodes), as well as the geometry of the multidentate organic ligands (linkers), determine the structure of the resulting materials. If the geometrical combination leads to a divergent arrangement, infinite coordination polymers or networks can be formed. A very prominent subgroup of these materials are the porous coordination networks - also called metal-organic frameworks (MOFs) - which have been designed for a multitude of different applications (Figure 1) 1, 2.

In contrast, if the geometrical combination allows for a convergent arrangement of nodes and linkers, discrete two- or three-dimensional structures - so called supramolecular coordination complexes (SCCs) - can be obtained (Figure 1) 3. By fine tuning the employed components, i.e. judicious choice of metal centers and complementary multidentate ligands, their size and (outer and inner) shape can be carefully controlled 4-7, which enables the synthesis of compound libraries. The two commonly applied synthetic strategies to obtain SCCs are the so-called edge- and face-directed approaches 5. The latter was developed by Fujita in the late 90s and is also referred to as the 'paneling method' 8. Hereby, the SCC is built up using planar multidentate ligand molecules, which upon coordination to (convergently oriented) vacant sites at metal nodes, form the faces of supramolecular polyhedra or polygons. The edge-directed approaches, pioneered by Stang *et al*. 4, 9, mainly employ bidendate 'banana-shaped' pyridyl-based ligands to form the edges of SCCs, mostly in combination with Pd(II) and Pt(II) metal nodes 7. Raymond *et al.* significantly progressed both approaches, extending the investigations towards the dynamic behavior 10 and chirality^11^ of SCCs, and catalysis in SCCs 12, 13. Nitschke *et al* introduced the concept of 'subcomponent self-assembly'^14^ according to which the actual linker is formed *in situ* (e.g. by imine formation out of aldehydes and amines) and which also allows for covalent post-assembly modifications of the SCCs 15. Recently, Fujita and coworkers reported the synthesis and characterization of 'Pd_48_L_96_', the largest discrete self-assembled edge-directed polyhedron obtained so far, demonstrating the scalability of the size of SCCs 16.

Although a variety of structures have been reported in the literature, SCCs have been exploited for diverse applications only in recent years. In fact, among the most attractive areas of applicability, catalysis 17, sensing and molecular recognition 18-20 are certainly the most explored. Interestingly, the three-dimensional SCCs as the metallacages and chiral helicates have received great attention in the biomedical context. In general, metallacages feature an internal cavity (as a class of metallocavitands^21^) accessible to guest encapsulation, and thus, exploitable for various functions and applications involving host-guest chemistry 12, 20, 22. Instead, helicates have been studied for their molecular recognition properties of nucleic acid structures, with possible applications in therapy 23.

Moreover, a recent area of considerable interest is the design and development of photoactive cages and capsules in which either the metal ion complexation or the bridging ligand are endowed with luminescence properties 24. Such cages provide both a high concentration of chromophores and defined cavities to govern the host-guest optoelectronic interactions, and can be exploited for the design of novel imaging agents, as well as for sensing and photoactivation in biological systems.

**Figure 2:**
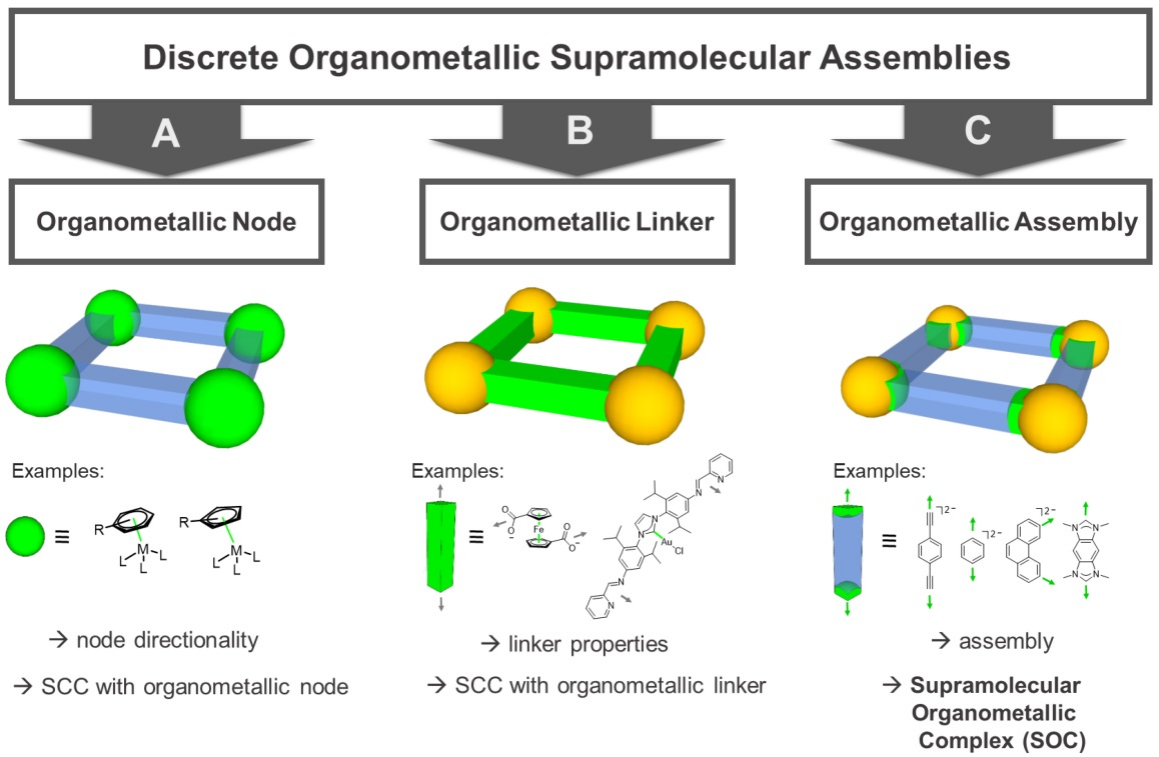
Classification of supramolecular organometallic assemblies into different groups: **(A)** Assemblies with organometallic nodes (i.e. carbon metal bond within metal node) 25-29. **(B)** Assemblies with organometallic linker molecules (i.e. carbon metal bond within linker molecule) 30, 31. **(C)** Supramolecular Organometallic Complexes (SOCs, i.e. assemblies with a carbon metal bond between node and linker) 4, 32, 33. Green: location of organometallic bond.

In addition to the SCCs built up of metal nodes and organic linkers by classic coordination chemistry using heteroatom donors, organometallic fragments featuring metal-carbon bonds have been introduced. Hereby, a carbon donor can in principle be implemented both in the linker molecules as well as in the capping ligands of the metal nodes (Figure 2). Concerning “organometallic nodes”, often very stable* η*^5^-(cyclopentadienyl) or *η*^6^-coordinated (benzene) metal half-sandwich or metal carbonyl complexes are employed (Figure 2A). In this case, the ligand is influencing the coordination geometry of the metal node, e.g. only allowing for a *cis*-coordination of potential linker molecules and therefore, determining the directionality leading to an edge-directed SCC assembly. Since the organometallic metal-ligand fragment is contained in the node, the assembly itself is still based on classic coordination chemistry. Examples of these compounds have been reported by various groups.^25^-^29^

There are two options for the implementation of the organometallic character into the linker molecules. First, if the carbon donor is not interacting with the metal nodes of the assembly but the organometallic bond is an integral part of the ligand molecule itself - rendering it an “organometallic linker” (Figure 2B) - the organometallic bond-formation is primarily influencing the linker stability, geometry and properties. Therefore, the organometallic bond is only indirectly affecting the self-assembly, which is again determined by classic coordination chemistry. In this case, the final compound can be also classified as a SCC. Examples of these kind of ligands used in SCCs are metallocene-based linkers, featuring more than one heteroatom as donor, e.g. ditopic ferrocenyl-dicarboxylic acids 31, tetratopic pyridyl-substituted Fe-metallocenes 34, or ditopic linkers featuring N-heterocyclic carbene (NHC) complexes 30.

Second, if the carbon donor is part of the linker-node connection, the (organic) linker is forming the organometallic bond to the metal node, thus, producing supramolecular organometallic complexes (SOCs) (Figure 2C). In contrast to the organometallic metal nodes, in this case, the carbon-metal bond is now structurally decisive for the “organometallic assembly” of the resulting SOC. Multidentate linkers used to build up such assemblies are mainly based on alkynyl, metal-arene 4, or NHC-donor groups. In particular, multidentate NHC ligands have undergone an enormous development in the past years, pioneered by the groups of Bielawski 35, Peris 36, and Hahn 37, also towards implementation in SOCs 32, 33. The possibility of tailoring the electronic and steric properties of the NHCs by synthetic modification of the heterocycle (e.g. so called backbone- or wingtip-modification) 38-41 also allows for the introduction of water-solubility 42 and therefore, renders these donors an excellent class for SOC linker design.

Furthermore (late transition metal) NHC complexes in general are very stable and - with few exceptions (e.g. Ag(I) complexes) - the ligands can be regarded as “spectator ligands” 43, i.e. they stick to the metal ion and do not dissociate or get transferred. As a result, there is a significant difference with respect to the SCCs based on classic coordination chemistry. The latter can be regarded as dynamic assemblies which in principle can reversibly form and disrupt. In contrast, late transition metal NHC-based SOCs in general can be regarded as 'static' structures, which do not reversibly assemble and disassemble. The strong metal-NHC bond (e.g. Au(I)-NHC bond) 44 disfavors a dissociation, which would lead to free N-heterocyclic carbene ligands. Free NHCs are furthermore very basic and therefore, likely to be protonated to form the corresponding azolium salt (especially under physiological conditions), which means they would be irreversibly removed from the dissociation equilibrium. This was deliberately used in the case of the pillarplexes, a family of NHC-based organometallic cavitands (a subclass of SOCs) featuring a tubular pore introduced in 2016 45. Interestingly, the pore can be used to selectively incorporate linear molecules, the pillarplexes can be easily made water soluble by simple anion exchange and the Au(I) congeners exhibit an intrinsic luminescence, without the necessity of further functionalization. Upon lowering of the pH value, the metal ions (in this case Ag(I)) can be released and the corresponding imidazolium ions are obtained, which was implemented in a mechanically interlocked molecular switch with two discrete states 46. It is worth mentioning that coinage metal NHC complexes themselves are potentially interesting in the biomedical context. Hereby, silver NHC compounds are known to possess anti-microbial activity 47 and gold NHC compounds have relevance as anti-cancer metallodrugs 48-50. Therefore, NHC-based SOCs are promising candidates for investigation towards biomedical applications, which was also shown in a very recent study on the toxicity of the pillarplexes 51.

Overall, this review is not intended to present details on the synthetic strategies to achieve various SCCs or SOCs, rather to summarize some general design principles focusing on the most recent examples from those systems having applications in anticancer therapy. Recent thematic reviews from different groups have commented the exponential progresses made in the design, synthesis and numerous applications of these discrete nanostructures 5, 6, 17, 20, 21, 52, and we refer the reader to these papers for further information. Here, we will focus on discussing SCCs as cytotoxic agents or as drug delivery systems for chemotherapics, emphasizing the high versatility and tunability of these scaffolds. In addition, we will present the few relevant *in vivo* studies on anticancer SCCs, which validate the concept and pave the way to their clinical application. Thus, we aim at providing the future outlook for this exciting research area, which, in defining the various challenges, will hopefully stimulate new ideas within the supramolecular, bioinorganic and medicinal chemistry communities.

## 2. SCCs as anticancer agents

Taking inspiration from the clinical success of the Pt(II) anticancer drug cisplatin 53, 54, SCCs themselves are under investigation as experimental cytotoxic anticancer agents. In the next sections, some of the most investigated systems are presented, including coordination and organometallic supramolecular systems, providing an idea of their main features and design principles.

### 2.1. Cytotoxic palladium and platinum SCCs

In palladium(II) and platinum(II) SCCs, the metal precursor can be *cis*-capped to allow only two coordination sites available for complexation to multidentate ligands to form discrete SCC architectures featuring square planar geometry around the metal centre 52. For example, 2D dinuclear Pt(II) and Pd(II) metallacycles coordinated to amide-based dipyridyl ligands and 1,1′-bis(diphenylphosphino) ferrocene ligands, have been studied as cytotoxic agents *in vitro* 55. Interestingly, both metallacycles displayed increased antiproliferative effects compared to their metal precursors and organic ligands, suggesting that the structure of the intact metallacycle is essential for the observed activity 55. Interestingly, the Pt(II)-based metallacycle was found to be a more potent inhibitor of cell proliferation against, head and neck, and thyroid cancer cell lines than cisplatin, and yet was less toxic against non-cancerous cells 55. The mechanism of action of both metallacycles was investigated *in vitro* against the T98G brain tumor cell line 55, The results show that both compounds are easily internalized by the cancer cells, and induce oxidative stress eventually leading to cell death by apoptosis.

Concerning 3D supramolecular architectures, a highly charged [Pt_6_L_4_]^12+^ metallacage was studied for its antiproliferative activity 56, and it displayed similar cytotoxicity range as cisplatin towards a range of human cancer cell lines, while it was ca. 7-fold less toxic than cisplatin towards normal lung cells. Moreover, the cage was found to be localized inside the cell nucleus using atomic absorption spectroscopy 56. It was also shown that the mechanism of action involves the compound's non-covalent binding to DNA *via* intercalation.

Studies by Crowley and coworkers on the effect of different ligands (functionalized trispyridyl scaffolds with rigid alkene linker *vs* benzotriazoles, hexane-triazoles and PEG-triazoles-bisfunctionalised phenyl rings) on the biological activity of Pd_2_L_4_ helicates have also been carried out, and showed a direct correlation between the stability of the helicate in biological media and its antiproliferative effects 57. Similar [Pd_2_L_4_]^4+^ (L = 1,3-bis-hexanetriazole phenyl) helicates endowed with sufficient stability in aqueous environment were also found to be up to seven-fold more toxic (IC_50_ ca. 6 μM) than cisplatin against the cisplatin-resistant MDA-MB-231 breast cancer cell line after 24 h incubation 57, and cell death appeared to be induced by disruption of the cell membrane 57.

**Figure 3:**
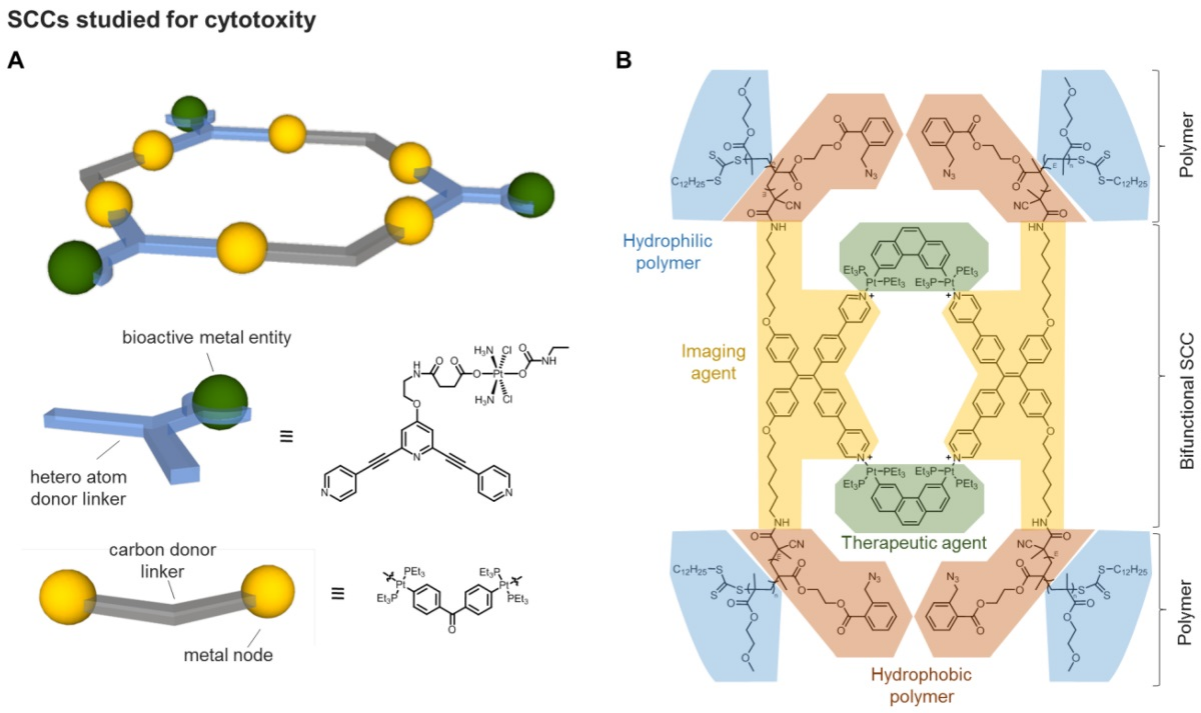
**A)** Schematic representation of a [Pt_3_L_3_]^6+^ hexagon *exo*-functionalised with three moieties of a Pt(IV) prodrug 58. **B)** The amphiphilic polymer** Pt-PAZMB-*b*-POEGMA**, containing glutathione (GSH)-responsive deblock copolymers as the arms and an aggregation-induced emissive Pt(II) metallacycle as the core unit 59.

Finally, examples of 3D metallacages, of the type Pd_2_L_4,_ have also been reported as experimental anticancer agents either *via* tethering or encapsulation of a cytotoxic agent, exploiting the so-called *prodrug* concept. As a recent example, the conjugation of prodrugs to the surface of a 2D SCC has been described. Specifically, a supramolecular [Pt_3_L_3_]^6+^ hexagon was formed by self-assembly between a dinuclear Pt(IV) precursor and a bidentate ligand conjugated to organoplatinum species. The resulting supramolecular hexagons would deliver three equivalents of cisplatin upon reduction of the Pt(IV) prodrug in the intracellular environment (Figure 3) 58. The antiproliferative effects of the supramolecular hexagon were tested against a range of cancer cell lines, sensitive or resistant to cisplatin, resulting in a more potent cytotoxic effect than cisplatin 58, even though no control with a Pt(IV) prodrug alone was reported. Mechanistic studies suggested that the prodrug induced apoptosis by causing DNA damage due to the intracellular release of cisplatin upon reduction of the Pt(IV) complex. Quantification of the intracellular Pt content suggested that the increased potency of the supramolecular hexagon was due to its higher cellular uptake compared to free cisplatin 58.

With the aim to exploit coordination-driven self-assembly to increase the anticancer potential of an adamantyl Pt(IV) prodrug, Lippard and coworkers used a tridentate ligand and a [Pt^II^(ethane-1,2-diamine)] precursor to achieve a cationic [Pt_4_L_6_]^12+^ cage able to encapsulate the Pt(IV) complex 60. Of note, the latter had low solubility in water (<500 μM) but became readily soluble when mixed with the cage. The hydrophobic adamantyl moiety of the prodrug molecule was postulated to be securely encapsulated within the hydrophobic cavity of the hexanuclear cage, as suggested by 1D and 2D NMR spectroscopy. The cage encapsulating the Pt(IV) complex was moderately cytotoxic in A549 human lung cancer cells, but still more potent than the Pt(IV) prodrug and of the hexanuclear Pt(II) cage alone 60. The mechanistic hypothesis is that the Pt(IV) complex is reduced intracellularly by ascorbic acid, thus, releasing cisplatin, 1-adamantylamine, and succinic acid, as suggested by NMR spectroscopy and mass spectrometry methods.

Further studies on a similar water soluble [Pt_4_L_6_]^12+^ metallacage, forming drug-loaded nanoparticles with an anionic polymer, revealed that the fluorophore fluorescein could also be encapsulated within the cavity 61. Thus, the fluorescein moiety was conjugated to a Pt(IV) prodrug and encapsulated in the metallacage, and the cellular uptake and release of the guest prodrug could be studied in HeLa cells *in vitro* by fluorescence microscopy 61. The necessity of optimizing the formulation of SCCs for drug delivery *via* formation of nanoparticles has also been elegantly addressed by Stang and coworkers using covalent conjugation of a polymer to formulate a Pt(II)-based supramolecular metallacycle into 50 nm nanoparticles, formed by self-assembly, for the delivery of doxorubicin (DOX) 59. The resulting **Pt-PAZMB-*b*-POEGMA** amphiphilic polymer is shown in Figure 3B. The impacts of the morphology and size of the supramolecular assemblies on their endocytic pathways, uptake rates and efficiency, as well as cytotoxic effects were also investigated *in vitro* 59. To evaluate the *in vivo* antitumor efficacy, HeLa tumor-bearing mice were intravenously injected with the DOX-nanoformualation (5.00 mg DOX/kg, 0.150 mg Pt/kg) and showed enhanced tumor reduction with respect to free DOX, while presenting low systemic toxicity 59.

### 2.2. Cytotoxic ruthenium(II)-arene SCCs

Biologically active Ru-based coordination and organometallic complexes 62 have recently prompted analogous studies of ruthenium(II)-arene supramolecular coordination complexes with a particular focus on their anticancer properties. Typically, ruthenium(II) ions (*d^6^* electronic configuration) adopt a hexa-coordinated octahedral coordination geometry. Hence, in order to produce discrete supramolecular entities, rather than extended coordination polymers or MOFs, an auxiliary ligand is needed to block some of the several Ru(II) coordination sites. In most cases, this is achieved by using dinuclear piano-stool ruthenium(II)-arene “clip” complexes linked together *via* pyridine containing multidentate ligands, to form a range of 3D and 2D supramolecular polyhedra 63-69. These metalla-assemblies possess different functional groups, situated either at the periphery or at the core of the assembly.

Supramolecular ruthenium(II) metallacycles were already reported in the late 1990's and displayed properties, such as water solubility and stability, which make them suitable for biological applications. It was initially postulated that cytotoxicity associated with supramolecular Ru(II) complexes was due to their intracellular dissociation, and subsequent binding of the released ruthenium cations to proteins and DNA, causing extensive cell damage and apoptosis 64. Interestingly, these complexes have been shown to cause cell death also by triggering excessive autophagy, the controlled process of recycling dysfunctional or destroyed proteins and organelles *via* lysosome digestion 70. Within a series of ruthenium(II)-arene metallarectangles with different paneling linkers, one derivative has been shown to be moderately cytotoxic *in vitro* against multidrug resistant human colon cancer cells (HCT-15/CLO2, IC_50_ ca. 16.5 μM) compared to cisplatin and doxorubicin 63, suggesting that the mechanism of cytotoxic action of these supramolecular structures is different from those of classical anticancer metallodrugs and requires further investigation.

Similarly to metallacycles, ruthenium(II)-arene metallabowls have also been developed and tested for their antiproliferative properties *in vitro* against a range of cancer cell lines (colorectal, gastric, and liver cancer cells) 69-71. Among them, a metallabowl featuring 8-dihydroxy-1,4-naphthaquinonato ligands was two-fold active than both cisplatin and doxorubicin against HCT-15 cells 69. Further investigations showed that, upon metallabowl exposure, the expression of two known colorectal cancer suppressors, p53 and the Adenomatous polyposis coli (APC) gene, increased in HCT-15 cells 69, 71.

By introducing tridentate, planar ligands to the binuclear arene Ru(II) “clips”, a range of hexanuclear 3D Ru(II) metallacages have been reported by variation of the paneling linker ([Ru_2_(*p*-*^i^*PrC_6_H_4_Me)_2_(*OO*∩*OO*)][CF_3_SO_3_]_2_ (*OO*∩*OO* = 2,5-dioxydo-1,4-benzoquinonato [dobq], 5,8-dihydroxy-1,4-naphthaquinonato (donq), and 6,11-dihydroxy-5,12-naphthacenedionato [dotq] etc.)) 72-75. Among them, the dinuclear “clip” containing donq as the bridging ligand was a moderate inhibitor of cell viability against a range of cancer cell lines (25 < IC_50_ < 90 μM), whereas all the other tested metallacages were non-cytotoxic 64, 76. Alongside ruthenium-arene complexes exhibiting antiproliferative effects *per se*, the synthesis of three new pyrenyl-containing dendrimers and their encapsulation into a water-soluble hexaruthenium(II)-arene metallaprisms was achieved, and the resulting host-guest systems were shown to possess enhanced antiproliferative effects in cancer cells *in vitro* with respect to the free components 77.

### 2.3. DNA Targeted SCCs

**Figure 4:**
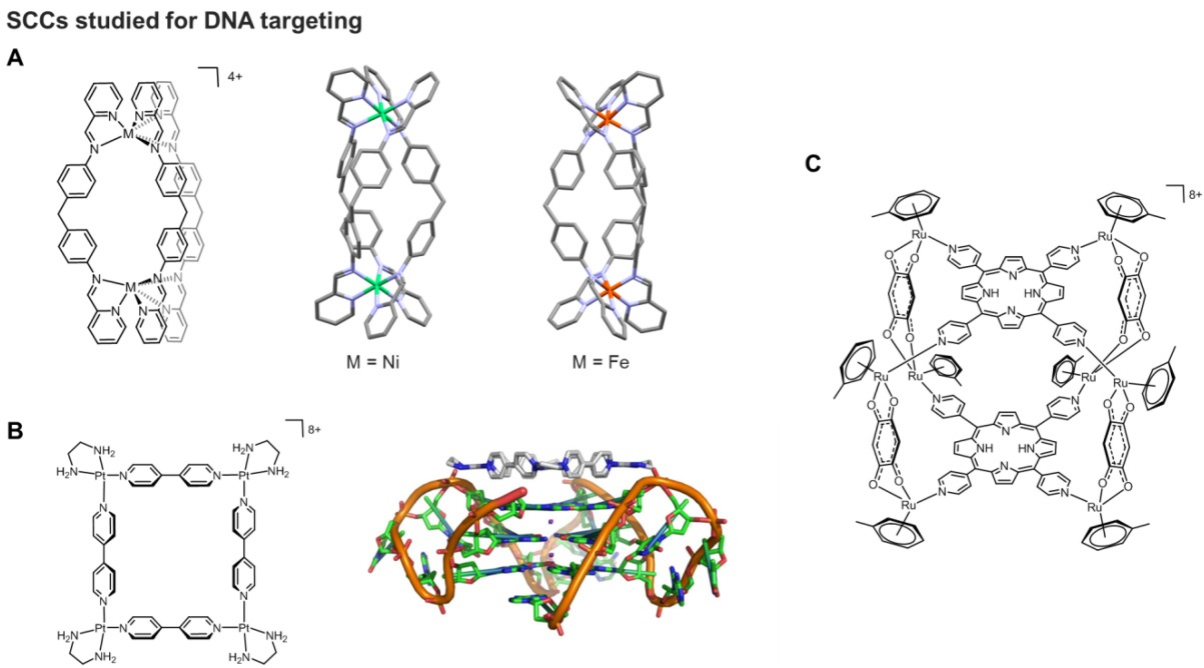
**A)** Schematic representation and corresponding X-ray structures of a cylindrical [Ni_2_L_3_]^4+^ helicate (CCDC n° 722438) 78 and a [Fe_2_L_3_]^4+^ helicate (CCDC n° 622770);** B)** Schematic representation of a multinuclear Pt(II) metallacycle acting as quadruplex binder and telomerase inhibitor and its adduct with a G4 structure studied by molecular modeling 92 (Adapted with permission from '*J. Am. Chem. Soc.* **2008**, *130* (31), 10040-10041'. Copyright 2008 American Chemical Society.); **C)** Schematic representation of a Ru_8_ cage bearing porphyrin ligands studied as nucleic acid binder 93.

Most of the numerous cytotoxic SCCs mentioned in previous sections were not designed to specifically target cancer cells, thus, leading to possible side effects. With the aim to develop tumor directed SCCs, the chirality of some of these scaffolds was exploited to direct molecular recognition of specific biological targets, namely nucleic acids. Within the helicates family, Hannon and coworkers developed the synthesis of 'cylinders', dinuclear triple-helical compounds which are prepared in a single step from a pyridyl-aldehyde, a diamine and an octahedral metal (usually Fe(II) or Ni(II) ) (Figure 4A) 78. The cylinders differ from earlier helicates also since they are endowed with certain rigidity along the length of the structure, due to π-stacking interactions between the rings of the diphenylmethane 'spacer'. By contrast, the Lehn helicate systems comprise bipyridine ligands linked by flexible alkyl or alkylether chains, introducing a higher degree of flexibility into the helical structure 79, 80.

A first study investigated the mode of binding of a binuclear Fe(II) triple-stranded cylinder to a DNA model by NMR spectroscopy and computational modelling techniques 81. The obtained results suggested that the cylinder binds to the DNA major groove. It was also revealed that although a racemic mixture of the chiral helicate was introduced to the double stranded oligonucleotide, only the *M*-enantiomer was able to bind DNA causing a change in its conformation 81. The mode of binding of the Fe(II) cylinder was further studied and revealed that the helix preferentially binds to short (8-10 base pairs) purine-pyrimidine tracts within the DNA sequence 82. The affinity for specific DNA sequences proved a promising feature to target cancer cells *via* binding of the helicates to oncogenes 82.

Later on, it was also discovered that Fe(II) cylinders have a high specificity for RNA 3-way junctions 62, as well as for certain non-canonical secondary DNA structures, such as DNA bulges 83, 84 and G-quadruplex DNA (G4) 85. In particular, targeting telomeric G4s and stabilization of these structures has been shown to inhibit telomerase activity, leading to cell death 86. To that end, a pair of enantiomeric Fe(II) helicates which were soluble in aqueous media were synthesized, and their affinity for human telomeric G4s was assessed 85. The *P*-enantiomer Fe(II) helicate was found to bind strongly and selectively to the G4, whereas the *M*-enantiomer showed no association. Furthermore, the strong binding affinity to G-quadruplex DNA translated into strong inhibition of telomerase activity.

Triple stranded “Y-shaped” junctions are an example of another non-canonical DNA structure which form during DNA transcription and replication, and whose regulation may allow to achieve antiproliferative effects and, most importantly, cell cycle control. Several studies have shown that these structures can be targeted by binuclear metallahelicates (Fe(II) and Ru(II)-based) 23, 82, 84, 87-90. In 2010, Hannon and coworkers showed that the stabilization of Y-shaped junctions by supramolecular Ru(II) cylinders severely inhibits the function of polymerase enzymes, accounting for their cytotoxicity 88. This study provides crucial evidence that the non‐covalent DNA binding of the cylinders can indeed affect the ability of proteins to process the DNA information. Further studies of DNA binding of Fe(II) helicates revealed that, to facilitate strong binding to the major groove of duplex DNA, a rigid helicate is preferred over the analogous flexible helicates 91. The former was also a potent cytotoxic agent against cisplatin resistant human ovarian carcinoma cells 91.

In addition to Fe-based helicates, the self-assembled platinum(II) molecular square [Pt(en)(4,4′-dipyridyl)]_4_ (en = ethylenediamine) has been reported to be an efficient G-quadruplex binder and telomerase inhibitor (Figure 4B) 92. Molecular modeling studies combined to molecular dynamics (MD) calculations suggested that the square arrangement of the four bipyridyl ligands, the highly electropositive nature of the overall complex, as well as hydrogen bonding interactions between the ethylenediamine ligands and phosphates of the DNA backbone all contribute to the observed strong binding affinity to the G4 (Figure 4B). More recently, a supramolecular [Pt_2_L_2_]^6+^ binuclear metallacycle with large, planar 2,7-diaza-pyrene-based ligands has been explored for its DNA binding properties 94. This interaction caused DNA bending, which in turn prevented DNA processing and replication. Moreover, the metallacycle exhibited antiproliferative effects in cancer cells and different spectrum of activity with respect to cisplatin 94. Supramolecular Pt(II) quadrangular boxes with L-shaped 4,4′-bipyridine ligands were also shown to bind duplex and G-quadruplex DNA motifs in a size-dependent fashion 95. Specifically, three dinuclear Pt(II) molecular squares of distinct size (ranging between 110-220 Å) inhibited cancer cells' growth and heavily influenced the expression of genes known to form G-quadruplexes in their promoter regions. Interestingly, the smallest Pt-box displayed less activity, but enhanced selectivity for the G4 promoter *c-Kit*, as shown by FRET (Fluorescence Resonance Energy Transfer) DNA melting assays 95.

Finally, 3D SCCs, including boxes and cubes, have been reported to be able to interact with nucleic acids. For example, porphyrin-based scaffolds were designed and linked *via* ruthenium complexes used as bridging blocks able to connect two porphyrin units and create octa-ruthenium supramolecular cubes (Figure 4C) 93. The G4 binding properties of the cubes were studied by different techniques, including fluorescence intercalation displacement (FID) and surface plasmon resonance, and the obtained results showed strong interactions with different G4 models, but also scarce selectivity with respect to duplex DNA.

### 2.4. Metalla-assemblies as PDT agents

In traditional photodynamic therapy (PDT), a photosensitizer interacts with oxygen to produce reactive oxygen species (ROS) upon light activation. ROS induce cell death *via* different pathways, including extensive damage of unsaturated lipids and certain amino acid side chains as well as of the nitrogenous bases of nucleic acids. PDT is considered a promising treatment as it possesses several benefits in comparison to common cancer therapies 96. However, this technique is still limited due to a number of drawbacks, generally associated with a residual photosensitivity after PDT treatment, difficulty to treat metastases, the need of light at a specific wavelength to be able to reach deeper tumors, as well as an adequate tissue oxygenation. For example, several ruthenium-arene metalla-assemblies coupled to photosensitizers, including porphyrins, were prepared and tested as PDT agents 97. Recently, Therrien and coworkers have synthesized an anthracene-based metalla-rectangle with the idea to improve the cell uptake of the photosensitizer (anthracene) 98. In the presence of oxygen and light activation, anthracene forms an endoperoxide intermediate. The endoperoxide formation is reversible, and oxygen can be released in a different environment. Unfortunately, upon formation of the metalla-assembly, the propensity of the anthracene moiety to react with oxygen to form endoperoxide derivatives was lost 98.

**Figure 5:**
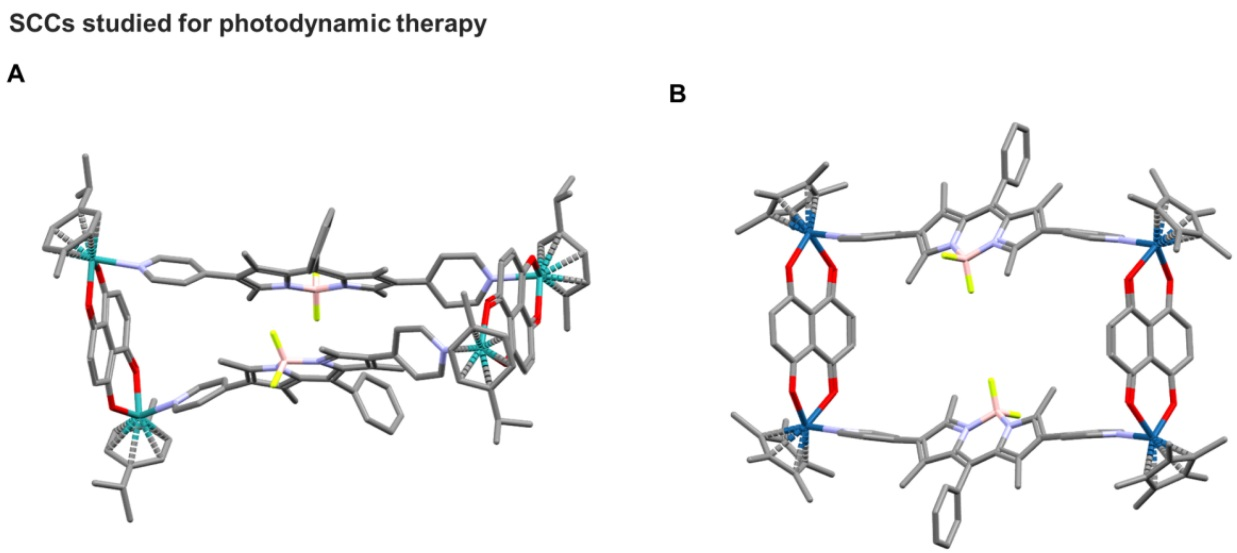
X-ray structures of rectangles (**A**) 2^4+^ and (**B**) 4^4+^ from ref. 105.

Noteworthy, in this area, Borondipyrromethene (BODIPY) is a class of fluorescent dyes used for many applications such as light harvesting, imaging, in solar cells and for PDT 99-101. Thus, BODIPY-based palladium, platinum, iron, and zinc supramolecular structures have been synthesized by self-assembly and analysed for their host-guest chemistry and biological properties.^102^-^104^ Inspired by these properties of BODIPY-based supramolecules, Gupta, Lee and coworkers designed, synthesized, and studied for their antiproliferative activities new ruthenium and iridium metalla-rectangles featuring a BODIPY-based linker (Figure 5) 103, 105. Confocal laser scanning microscopy studies suggested that the compounds use a cytoplasmic mechanism of action in causing cell death. Additionally, binding studies revealed the ability of compounds to interact with both DNA and protein 105. However, the possibility of using such metalla-rectangles as PDT agents remains to be explored.

One of the first examples of a SCC featuring a BODIPY ligand for applications as *theranostic* agent was reported by Cook and coworkers 106, who investigated the coordination-driven self-assembly of two novel Pt(II) supramolecular triangles containing a pyridyl-functionalized BODIPY ligand. While the Pt(II) moieties can also act as cytotoxic agents, the BODIPY cores within triangles enable their transport within cancer cells to be visualized by confocal laser scanning microscopy. Moreover, the BODIPY ligands also form the basis for use as a photosensitizer for photodynamic therapy (PDT). The obtained results showed that the combination of PDT and chemotherapy greatly enhances anticancer efficacy through a synergistic therapeutic effect, particularly against drug resistant cancer cells *in vitro* 20.

## 3. SCCs as drug delivery systems for anticancer agents

The focus of this chapter is on SCCs that show promise for biomedical applications not due to their intrinsic anticancer potential, but for their favorable properties as drug delivery systems. This is particularly relevant to cancer chemotherapy, whose success rate remains limited, primarily due to scarce selectivity of drugs for the tumor tissue, often resulting in severe toxicity and in the development of drug resistance. So far, lipid nano-systems, such as liposomes and micelles, along with virus-inspired vectors and polymeric particles, dendrimers as well as inorganic nanoparticles, have been studied to deliver bioactive compounds to tumor sites. However, such targeted constructs have several limitations: for example, polymers and dendrimers often require considerable synthetic effort and can be plagued by low yields and largely amorphous final structures, while nanoparticles often present issues of toxicity and lack of biodegradability 107.

In this context, supramolecular metallacages feature a number of properties that make them attractive candidates for future drug delivery systems. For example, the rigid, porous structure offers a secure cavity for small drug molecules, to protect them from metabolism, and the ability to modify the ligand structure both pre- and post- self-assembly allows for the properties of the resulting cage to be improved. Furthermore, since metallacages, at variance with MOFs, are discrete chemical entities, the issues of solubility in an aqueous environment can be potentially overcome. Despite these attractive features, SCC drug delivery is still in its infancy 108.

Overall, SCC as drug delivery systems can be based on *i)* both encapsulation of a drug, driven by hydrophobicity of the cargo drug molecule and the host cavity, and non-covalent interactions within the host cavity (e.g. H-bonding, van der Waals), as well as *ii)* covalent bonding of a prodrug species to the SCC architecture. In the latter case, the prodrug can then be cleaved and activated *via* external chemical stimuli to allow drug release in a controlled manner. In this chapter, representative examples of both strategies are provided.

**Figure 6:**
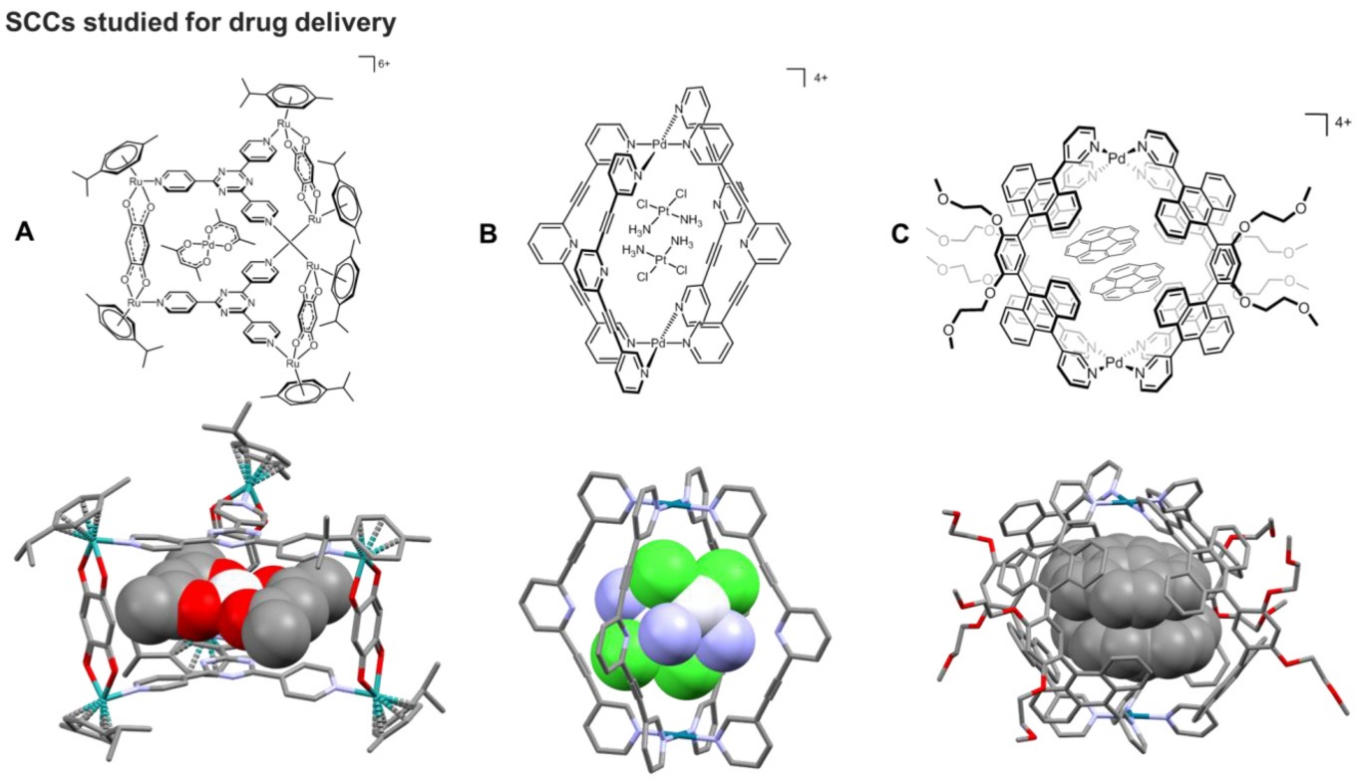
**A)** Schematic representation and corresponding X-ray structure (CCDC n° 673229) of a [[Ru_2_L']_3_L_2_]^6+^ cage encapsulating [Pt^II^(acac)_2_] (acac = acetylacetonato) 29. **B)** Schematic representation and corresponding X-ray structure (CCDC n° 853227) of an *exo*-functionalised [Pd_2_L_4_]^4+^ metallacage encapsulating two equivalents of cisplatin 111. **C)** Schematic representation and corresponding X-ray structure (CCDC n° 902397) of a [Pd_2_L_4_]^4+^ capsule encapsulating two equivalents of corannulene 112.

Based on previous studies on ruthenium(II) metallacycles in 2008 109, pioneering work by Therrien and coworkers on SCCs as drug delivery systems focused on the study of water soluble hexaruthenium metallacages (metalla-prisms) able to encapsulate lipophilic molecules.^29^ Thus, the cationic hexanuclear metalla-prism [(*p*-cymene)_6_Ru_6_(tpt)_2_(dhbq)_3_]^6+^ (tpt = 2,4,6-trispyridyl-1,3,5-triazine; dhbq = 2,5-dihydroxy-1,4-benzoquinonato) was shown to encapsulate two hydrophobic Pd^II^ and Pt^II^ complexes [M(acac)_2_] (M = metal, acac = acetylacetonato) (Figure 6) 29. While the [M(acac)_2_] complexes are completely inactive due to their inherent lack of solubility in water, the metalla-prism is water soluble and moderately cytotoxic (IC_50_ ca. 23 μM) against human ovarian A2780 cancer cells. This initial study provided the proof-of-concept for the so-called “*Trojan horse strategy*” of protecting a cytotoxic agent in the cavity of a metallacage until, after cell uptake, the drug can be released and perform its cell-killing activity. In fact, the encapsulated [Pd(acac)_2_] - ([Pd(acac)_2_]⊂[(*p*-cymene)_6_Ru_6_(tpt)_2_(dhbq)_3_]^6+^]) - was 20-fold more cytotoxic (IC_50_ ca. 1 μM) than the empty metalla-prism 29.

Following these promising results, a hexaruthenium metallacage of the type [Ru_6_(*p*-*i*PrC_6_H_4_Me)_6_(tpt)_2_(C_6_H_2_O_4_)_3_]^6+^ was investigated for the release mechanism of encapsulated fluorescent pyrene derivatives and for its anticancer properties *in vitro* 28. The obtained results showed that, while the free pyrene derivative and the cage complex alone were scarcely cytotoxic (IC_50_ ca. 16 µM or higher), the host-guest complex was considerably more active (IC_50_ ca. 6 µM) 28. The increased cytotoxicity of the water soluble cage-pyrene complex was due to an increased uptake of the overall system as shown by fluorescence microscopy. Of note, the fluorescence of the pyrene derivatives is quenched upon encapsulation, allowing for the release of the molecule to be monitored by fluorescence spectroscopy 28. In further studies, the encapsulation properties of the hexaruthenium metallacage with a series of functionalized fluorescent pyrene derivatives was characterized using NMR (^1^H, 2D, DOSY) spectroscopy and electrospray ionization mass spectrometry (ESI-MS) 110. The antiproliferative properties of the vacant cage and the pyrene-cage complexes were studied in A2780 ovarian cancer cells, and the host-guest complexes showed the lowest IC_50_ values 110. The study also demonstrated that the hexaruthenium cage complexes can improve the efficacy of insoluble inhibitors *in vitro.*

The effect of the portal size of the hexaruthenium metallacage complex on the retention of the planar guest molecules, [Pd(acac)_2_] complex and 1-(4,6-dichloro-1,3,5-triazin-2-yl)pyrene, was also investigated 113. Thus, three hexaruthenium cages were prepared by extending the polycyclic aromatic system in the di-ruthenium bridging ligands, using the 1,4-naphthoquinonato, 1,4-anthraquinonato, and 5,12-naphthacenedionato analogues, which progressively decreased the portal size of the cage, while the internal cavity remained largely the same. The host-guest properties of these water-soluble supramolecular drug delivery systems were studied in solution by NMR and fluorescence spectroscopy. The results showed that the complex with the largest pore size (estimated to be approximately 7.4×10.2 Å by molecular modeling) is more stable, suggesting that a larger pore size facilitates the entrance of the guest molecule in the cage, while the smaller pore size retains the guest molecule more effectively 113. Inductively Coupled Plasma Mass Spectrometry (ICP-MS) and fluorescence microscopy allowed to assess that all cages deliver the host to intracellular organelles and the mechanisms of uptake involve endocytosis/macropinocytosis rather than passive diffusion across the cell membrane 113.

Other examples of SCCs as drug delivery systems, based on other transition metals, include surface functionalized porous coordination nanocages of Cu(II) and 5-(prop-2-ynyloxy)isophthalic acid (pi), bearing a water solubilizing polymer (PEG5k), which were synthesized using a “click chemistry” approach 114. The scaffold is composed of 12 di-copper paddlewheel clusters and 24 isophtalate moieties, with 8 triangular and 6 square windows that are roughly 8 and 12 Å across, respectively. The internal cavity has a diameter of ca. 15 Å and the cage has high stability in aqueous medium. In addition, the cages' drug loading and release capacity has been evaluated using the anticancer drug 5-fluorouracil (5-FU) 114. Drug release experiments were carried out by dialyzing the drug-loaded Cu(pi)-PEG5k against phosphate buffered saline (PBS) solution at room temperature. Interestingly, around 20% of the loaded drug was released during the first 2 hours, while a flatter release curve can be observed up to 24 hours. The slow release has been associated to the slow diffusion rate of 5-FU caused by the strong interaction between Lewis acid sites in Cu(pi) and basic site of 5-FU.

Based on previous work by Fujita and coworkers 115, and within the M_2_L_4_ cage family, Crowley *et al.* designed a cationic [Pd_2_L_4_]^4+^ cage using (2,6-bis(pyridin-3-ylethynyl)pyridine) as the bidentate ligand, and characterized the 3D system by various methods, including ^1^H NMR spectroscopy, ESI-MS and XRD 111. Interestingly, the encapsulation of the anticancer drug cisplatin within the metallacage cavity, lined with the nitrogen atom from the central pyridine of the ligand, was demonstrated by XRD studies, revealing that two molecules of the drug could be contained (Figure 6) 111. The release of cisplatin was facilitated by the introduction of competing ligands (4-dimethylaminopyridine or Cl^-^) to disassemble the cage, as shown by ^1^H NMR and ESI-MS. Unfortunately, while the cisplatin-cage host-guest complex formed in acetonitrile and DMF, in more hydrogen bond competitive solvents (water and DMSO) no host-guest interaction was observed 116. Additionally, the parent Pd(II) based cage decomposed rapidly in the presence of nucleophiles. In fact, the same group demonstrated that the stability of [Pd_2_L_4_]^4+^ cages when exposed to biological nucleophiles highly depends on the ligand structure: specifically, triazole-based ligands were found to lead to significantly more stable metallacages in comparison with pyridine-based scaffolds 57.

More recently, Casini and coworkers explored similar cationic [Pd_2_L_4_]^4+^ systems featuring bis(pyridyl) ligands - of general scaffold 3,5-bis(3-ethynylpyridine)phenyl) - and developed the *exo*-functionalization of the ligand to add different groups, including fluorescent tags facilitating the study of the cellular accumulation of these systems by fluorescence microscopy 117, 118. Structural studies by ^1^H NMR ad XRD were performed demonstrating encapsulation of cisplatin (Figure 6) 119. Notably, most of the reported metallacages and their precursors were non-toxic in healthy rat liver tissue *ex vivo,* making them suitable for application as drug delivery systems 120. Furthermore, the cytotoxicity of the [Pd_2_L_4_]^4+^ cages have been tested *in vitro* against a small panel of human cancer cells, showing scarce or moderate antiproliferative activities depending on the ligand scaffold 120. More importantly, the activity of encapsulated cisplatin in the benzyl alcohol-*exo*-functionalized Pd(II) cage was evaluated against SKOV-3 ovarian cancer cells, showing a marked increase in cytotoxic potency (IC_50_ = 1.9 ± 0.5 μM) compared to free cisplatin (IC_50_ = 15.4 ± 2.2 μM) and the vacant cage complex (IC_50_ = 11.6 ± 1.7 μM).^120^ Interestingly, the aromatic and highly conjugated ligands confer the [Pd_2_L_4_]^4+^ cages fluorescence properties, which allowed to study their uptake in cancer cells by fluorescence microscopy.

Water solubility and stability under physiological conditions are both crucial for the biological application of SCCs. Unfortunately, [Pd_2_L_4_]^4+^ cages of this type are scarcely soluble in water, despite their positive charge. Thus, there are different strategies to introduce water-solubility to the SCCs. The most straight-forward approach is a simple anion exchange, since the SCCs usually are positively charged complexes. It was shown, that the hexafluorophosphate salts of the aforementioned organometallic pillarplex SOCs, which are soluble in organic solvents like acetonitrile or dimethylformamide, can be easily converted into the corresponding acetates, which are extremely well water-soluble (> 1g / mL water) 45. In case an anion exchange is not applicable or not leading to the desired effect, covalent modifications at the linker molecules can be performed to introduce polar functional groups, e.g. sulfonates 121. Another strategy to increase the hydrophilic character of these systems has been demonstrated *via* the introduction of water soluble moieties in their scaffold, including PEG 122.

The control of the host-guest properties of the cavity defined by the SCC is another essential feature to implement them for drug encapsulation. For example, anthracene-based Pt(II)- and Pd(II)-linked coordination capsules provide a characteristic spherical cavity - with a diameter of ca. 1 nm and a volume of ca. 600 Å^3^ - contoured by polyaromatic frameworks (Figure 6) 112, 123, and that can accommodate various neutral molecules, through hydrophobic and π-stacking interactions, in aqueous solution 112, 123, 124. Fluorescence microscopy studies allowed investigation of the intracellular accumulation of the capsules 124. However, these systems, even without their guest molecules, manifest very pronounced cytotoxic effects, which make them unsuitable for drug delivery. Interestingly, the observed trends in the anticancer activity of the capsules and their host-guest complexes correlate with their different stabilities toward glutathione, estimated by NMR-based kinetic experiments 124. The data suggest the glutathione-triggered disassembly of the capsular structures in cells as a potential activation pathway for their cytotoxic activity.

For supramolecular metallacages, such as [Pd_2_L_4_]^4+^, with a molecular weight of ca. 2-3 kDa and diameter of ca. 10-15 Å, passive tumor targeting *via* the enhanced permeability and retention (EPR) effect is not likely to influence their delivery 125. Furthermore, it should be mentioned that the success in preclinical *in vivo* studies of drug accumulation in tumors due to the EPR effect has so far not translated into success in clinical trials 126. In this context, active tumor targeting mechanisms are crucial to achieve selectivity of metallacages for cancerous cells, for example *via* the conjugation of cancer-cell-specific ligands. However, this concept has been scarcely explored so far and few examples of bioconjugated cages are available in the literature. One study showed non-covalent peptide coating on self-assembled M_12_L_24_ coordination spheres 127, while encapsulation of a protein within a Pd_12_L_14_ cage has been achieved by appropriate *endo*-functionalization of the ligands 128. Of note, the latter was the first example of encapsulation of a protein within synthetic host molecules which may unveil new strategies to deliver proteins at specific site and to control their function 128. In this latter example, ligands were first tethered to the protein and then the cage was reconstituted *via* self-assembly upon addition of other ligands and metal precursors.

Within this framework, in 2017 Casini and coworkers reported on the first example of bioconjugation of self-assembled [Pd_2_L_4_]^4+^ cages *via* amide bond formation between the -COOH (or -NH_2_) *exo*-functionalized ligand/cage and a complementary residue on a model linear peptide 129. Certainly, other types of *exo*-functionalization, other than amide bond formation, for tethering metallacages to peptides or antibodies should be investigated, including click chemistry approaches 130. Afterwards, in a proof-of-concept study, the same group reported on [Pd_2_L_4_]^4+^ cages conjugated *via* amide bond to four ligands selective for binding to integrins αvβ3 or α5β1 131. The conjugated cages were first studied for their integrin recognition properties using an ELISA assay, and were demonstrated to maintain high binding affinity and selectivity. Cage formation and encapsulation of cisplatin was proven by ^1^H NMR, ^1^H DOSY and ^195^Pt NMR spectroscopy. Upon encapsulation, cisplatin showed increased cytotoxicity *in vitro*, in melanoma A375 cells overexpressing αvβ3 integrins, while it was not active against A549 human lung cancer cells, not expressing this specific integrin 131. Moreover, *ex vivo* studies in tissue slices indicated reduced toxicity towards healthy liver and kidney tissues for cage-encapsulated cisplatin. The reason for such reduced toxicity is that the encapsulated drug is less accumulated in these organs compared to the 'free' one, as demonstrated by the analysis of metal content by ICP-MS 131. It should be noted that anchoring of the peptide to the metallacages also allowed to substantially increase their solubility in aqueous environment. In general, it is worth mentioning that targeting supramolecular metal-based complexes to the desired site is essential to control off-target effects of the delivered chemotherapic agent, as well as to reduce the possible intrinsic systemic toxicity of the supramolecular metal-based drug delivery system.

## 4. *In vivo* activity of anticancer and diagnostic SCCs

**Figure 7:**
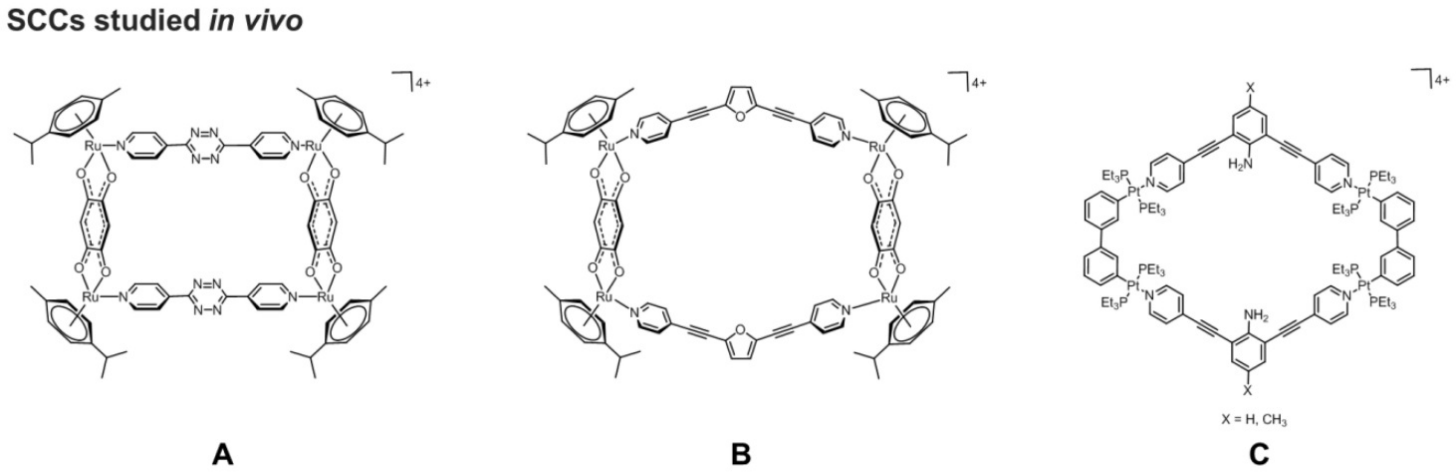
Schematic representations of **A)** two arene-Ru(II) metallacycles (left: 2D rectangular geometry; right: 'metalla-bowl' geometry)^70^ and of **B)** a rhomboidal Pt(II) metallacycle,^132^ studied *in vivo* for their anticancer properties.

Although the field of supramolecular coordination complexes as anticancer therapeutics is still in its infancy, a few preliminary *in vivo* experiments have been carried out. For example, the anticancer activity of two cytotoxic Ruthenium(II)-arene metallacycles, one with a 2D rectangular geometry and one featuring a metallabowl geometry (Figure 7A), was studied *in vivo* 70, using a hollow fiber assay, whereby a semipermeable fiber impregnated with the human colorectal carcinoma HCT-15 cells were implanted into the intraperitoneal and subcutaneous compartments of nude mice 70. The two ruthenium-arene SCCs were then administered to the impregnated nude mice, and the animals were left for 7 days before the hollow fibers were removed and the tumors examined. The study revealed that the metallabowl-type metallacycle was a more potent inhibitor of cancer cells growth than the metallarectangle. However, both these ruthenium-arene scaffolds were not as effective inhibitors of cell proliferation as cisplatin in the hollow fibers located in the intraperitoneal and subcutaneous regions of the host mice 70. The mechanism of induced cell death was investigated and the study revealed that both metallacycles induced autophagy in HCT-15 cells, and again the metallabowl was more potent than the metallarectangle, in line with the observed enhanced anticancer activity 70.

Concerning platinum(II) SCCs, in addition to the above mentioned **Pt-PAZMB-*b*-POEGMA** amphiphilic polymer by Stang and coworkers 60, a luminescent 2D Pt(II) metallacycle of rhomboidal geometry has also been studied *in vitro* and *in vivo* for its anticancer activity (Figure 7B) 132. Of note, the compound remained intact upon cellular internalization and did not photobleach under the conditions of the confocal microscopy experiment. Preliminary *in vitro* studies against lung (A549) and cervical carcinoma (HeLa) cells confirmed rapid cellular uptake of the platinum metallacycle 132. Afterwards, a mouse tumor xenograft model, generated using nude mice injected in the subcutaneous region with MDA-MB-231human breast cancer cells, was selected for the *in vivo* study. The tumors were allowed to reach a volume of 200 mm^3^ before drug administration. Mice were treated with a solution (300 μL) of Pt-metallacycle at a concentration of 0.6 mg/mL, administered v*ia* intraperitoneal injection every 3 days for 30 days. The study revealed that at the end of the treatment, a 64% median tumor volume reduction was observed in treated mice with respect to controls. Furthermore, the tumor growth inhibition, measured by the change in volume of the tumor throughout the length of the experiment and defined by the T/C ratio (in %, corresponds to the ratio between the Treatment (T) over the Control (C)),^133^ was calculated as 36%, well below the National Cancer Institute standard (as the lower threshold for tumor inhibition of <42%)) 134.

**Figure 8:**
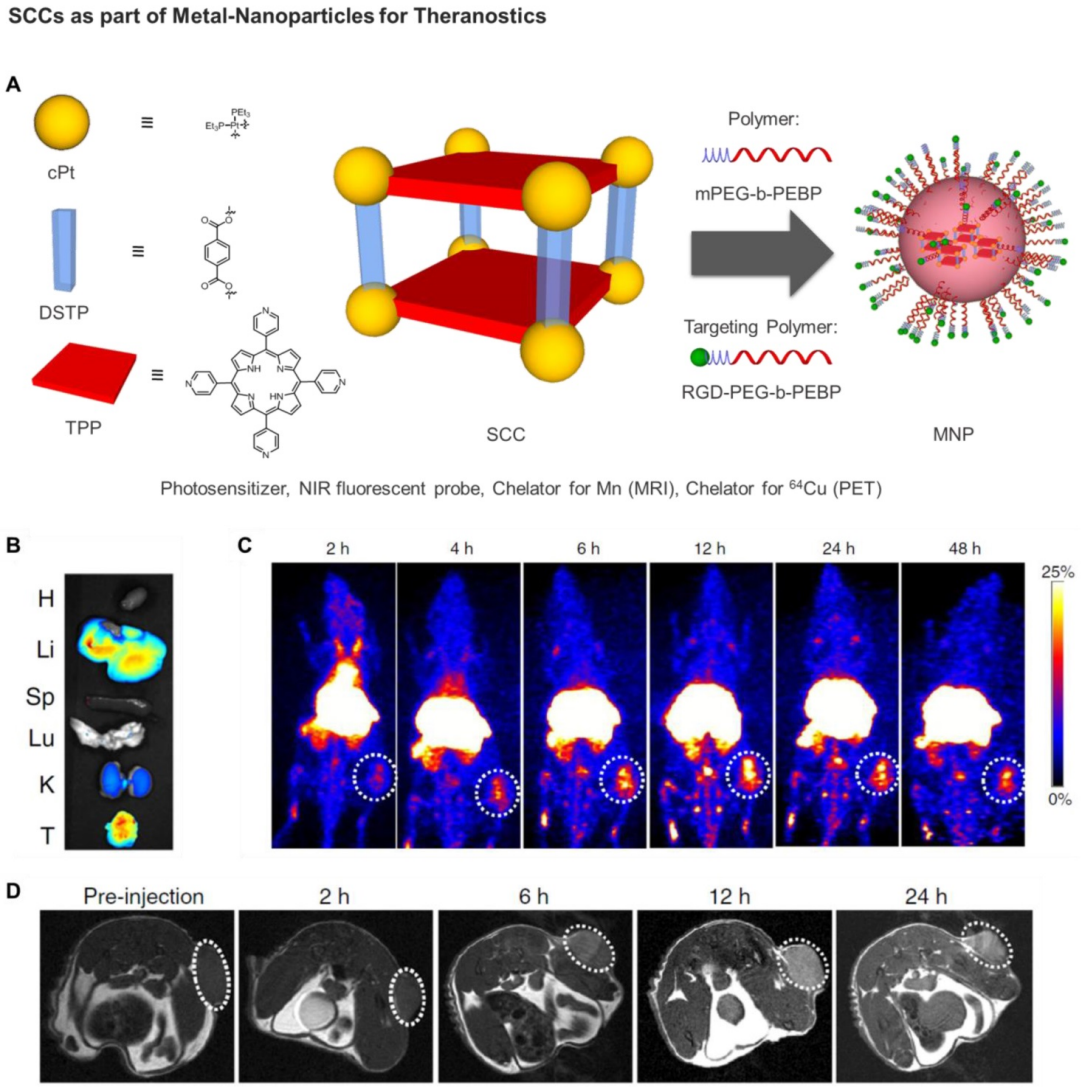
** A)** Schematic diagrams of the MNPs serving as a multifunctional theranostic platform. Structures of TPP, *c*Pt, DSTP, M, mPEG-b-PEBP, and RGD-PEG- b-PEBP 135. **B)** *Ex vivo* image of the main organs separated from U87MG tumor-bearing mice at 24 h post injection of MNPs. **C)** PET image of U87MG tumor-bearing nude mice at 2, 4, 6, 12, 24 and 48 h post injection of ^64^Cu@MNPs (150 μCi). The white circle denotes the tumor site. **D)** *In vivo* T_1_-weighted axial MRI images (7T) of the mice pre-injection and after injection of Mn@MNPs. The white circle denotes the tumor site. (Adapted with permission from '*Nature Comm.* **2018***, 9,* 4335-4335', licensed under a Creative Commons Attribution 4.0 International License. Copyright 2018 Springer Nature Publishing AG.)

Very recently, Chen, Stang and coworkers developed a sophisticated porphyrin-based metallacage through multicomponent coordination-driven self-assembly, acting as a theranostic platform to fabricate metal nanoparticles (MNPs) 135. In details, a discrete platinum(II) metallacage was synthesized, using therapeutic *cis*-(PEt_3_)_2_Pt(OTf)_2_ (*c*Pt), 5,10,15,20-tetra(4-pyridyl)porphyrin (TPP) and disodium terephthalate (DSTP) as the building blocks, with the idea achieving synergistic anticancer efficacy (Figure 8). Of note, both the fluorescence emission and ^1^O_2_ generation quantum yield of the porphyrins were dramatically increased upon formation of MNPs, which was favorable for both NIRFI and PDT 135. Furthermore, the nano-formulation was functionalized by two amphiphilic diblock polymers (mPEG-b-PEBP and RGD-PEG-b-PEBP). The resultant MNPs exhibited long blood circulation time and high tumor accumulation benefiting from the EPR effect and active targeting ability. Indeed, superior tumor suppression with respect to separate cisplatin treatment and light irradiation was realized without recurrence after single-dose injection of the targeted MNPs in xenograft models of tumors from αvβ3 integrin overexpressing U87MG cells and cisplatin-resistant human ovarian cancer A2780cisR cells 135. Moreover, in order to verify the *in vivo* anti-tumor performance and anti-metastasis effect of photochemotherapy, 4T1 (breast cancer) tumors were orthotopically inoculated in the mammary fat pads to produce spontaneous metastases in the lung, which was an experimental animal model for stage IV human breast cancer.

Notably, the combination of chemotherapy and PDT using the supramolecular MNPs exhibited superior anti-tumor efficacy with a 93.5% reduction in tumor volume, with respect to the least effective, and undergoing tumor recurrence during treatment, separate chemotherapic and PDT treatments 135. Excellent anti-metastatic effect was also achieved, which was attributed to the synergistic photochemotherapy. In addition, by chelating a positron emitting metal ion (^64^Cu) or a paramagnetic Mn ion, the ^64^Cu@MNPs (or Mn@MNPs) were shown to be excellent PET imaging and MRI agents, allowing precise diagnosis of tumor and real-time monitoring of delivery, biodistribution and excretion of the MNPs (Figure 8) 135. In fact, significant tumor accumulation was clearly visible in the U87MG tumor-bearing mice administered with MNPs at 6 h post injection, and exceptionally intensive signal was visible in the tumor area for more than 24 h in comparison with other tissues. Overall, these exciting preliminary *in vivo* studies highlight the potential of SCCs as anticancer agents and constitute the blueprint of next generation nanomedicines.

**Figure 9:**
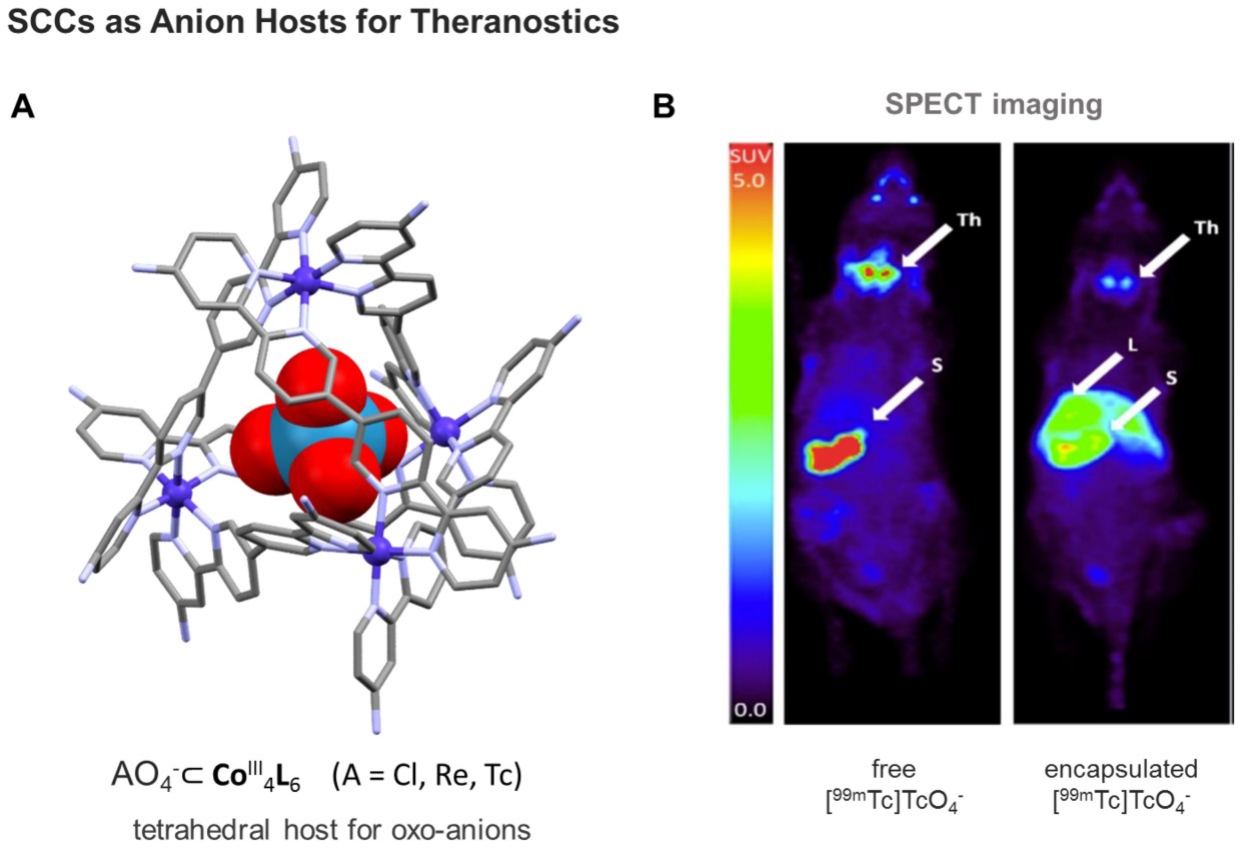
** A)** Structure of anion-binding Co^III^_4_L_6_ cages shown exemplarily at the X-ray structure of a tetrahedral SCC, featuring an encapsulated ReO_4_^-^ (CCDC n° 1864366) 136; **B)** Comparison of free [^99m^Tc]TcO_4_^-^ uptake in naïve mice (left) *vs* SCC-encapsulated [^99m^Tc]TcO_4_^-^ (right) monitored by SPECT imaging 136. Encapsulation results in reduced thyroid and stomach uptake, and increased liver uptake. Images are maximum intensity coronal projections. S = Stomach, Th = Thyroid, L = Liver. (Adapted with permission from '*J. Am. Chem. Soc.* **2018,** *140*, 16877-16881'. Copyright 2018 American Chemical Society.)

Finally, using a kinetically robust Co^III^_4_L_6_ (L = tetrahedron), Lusby and Archibald and coworkers showed the feasibility of encapsulating the γ-emitting [^99m^Tc]TcO_4_^-^ anion - the most widely used precursor in clinical nuclear diagnostic imaging - under conditions compatible with *in vivo* administration 136. Subsequent single-photon emission computed tomography (SPECT) imaging of the caged-anion revealed a marked change in the biodistribution of the host-guest system compared to the thyroid-accumulating free oxo-anion (Figure 9). While this example still needs optimization, including targeting to disease tissues, the obtained results move clinical applications of (metallo)supramolecular species a step closer.

## 5. Summary and Future Perspectives

During the past decade, increasing interest has been manifested in the design and synthesis of discrete metallo-supramolecular architecture for biomedical applications. Certainly, the examples reported in this review show that SCCs can be designed to feature an inherent bioactivity, *i.e.* to act as a cytotoxic metallodrug itself. There are several possible ways to include bioactivity in SCCs, either by the metals (e.g. upon metal release / disassembly of the SCC / photoactivation) or by biological activity of the organic linker molecule. Also, the structural prerequisites and the outer shape of the SCCs can significantly determine their bioactivity as was shown for the metal helicates. The latter, due to their tetracationic charge and cylindric shape, can ideally interact with negatively charged biomolecules as DNA, and thanks to their intrinsic chirality, even recognize very selectively special structures like three-way junctions 137-143. In addition, more complex architectures such as the interlocked metallacages reported by Clever *et al.* show a comparable interaction with DNA biomolecules 144. Overall, these examples hold promise to achieve targeting by molecular design.

In this context, the exact determination of the bioactive species (*speciation*) is very important, since for the modular built SCCs all individual components (metal ions, organic linkers), as well as decomposition products, can be potential cause of the observed activity. Therefore, the fine-tuning of the complex stability by linker-design (e.g. by using different donor moieties for linkers) in combination with the use of different (biologically active) metals are promising parameters for selectively influencing the bioactivity.

Indeed, when developing supramolecular metal-based complexes for biomedical applications one should control their stability in physiological media and in the presence of biologically relevant nucleophiles or reducing agents 57, 132. In general, the use of first row transition metals with prominent kinetic lability with respect to ligand exchange reactions, is not ideal to achieve robust systems in solution. Therefore, late transition metals such as Ru(II), Pt(II) and Pd(II) should be preferred. However, we envisage that the use of organometallic scaffolds, as in the case of SOCs, may be further exploited to increase the stability in biological environment of the resulting supramolecular systems. This feature would also be advantageous to reduce possible side-effects *in vivo*, including systemic toxicity due to off-target effects and extensive metal speciation. Within this framework, water-soluble NHC-based SOCs like the pillarplexes hold promise for application as metallodrugs (NHC complex character), drug-delivery systems (porous character) and diagnostic handles (in-built functionality: e.g. luminescence). Furthermore, this unique combination of properties renders organometallic supramolecular systems as highly potential candidates to investigate towards theranostic applications.

In the previous sections, we have also emphasized the potential of 3D-metallacages as drug delivery systems for anticancer agents. In fact, cages have distinct advantages over conventional container molecules based on covalent bonds (e.g. nanotubes and polymeric nanoparticles), including the synthetic ease with which the coordination cages can be self-assembled, the availability of a large library of building blocks, and the possibility to design selective guest encapsulations. Despite these advantages, in order to develop the full potential of SCCs for biological applications, several issues need to be addressed. Firstly, the solubility of most 3D-SCCs in water or physiological media is generally low. The only example of fully water-soluble construct *per se* (i.e. without additional water-soluble functionalities anchored) is the family of ruthenium-based prisms obtained by face-directed self-assembly, which was exploited to encapsulate and solubilize hydrophobic guests 75, 110, 145, 146. Regarding palladium(II)-based SCCs, the solubility of the final cages is often a real challenge, despite the multiple positive charges carried by the constituting metal ions. In rare examples where effort was put into varying the counter-ions and increasing the water solubility of the ligands by anchoring small water-soluble biomolecules, the final cages remained insoluble 147-149. Instead, bioconjugation of [Pd_2_L_4_]^4+^ cages to targeting peptides made the systems well water soluble 131.

The study of the drug encapsulation and release properties in SCCs is a key step in the validation of these systems for drug delivery. Understanding the driving forces of the host-guest chemistry is also pivotal to the optimization of the cage scaffolds. Various factors are already known to play a role, including possible interactions of the guest molecule with the host cavity (e.g. H-bonding, van der Waals, coordination and electrostatic interactions) as well as energetically favorable solvent rearrangement during guest desolvation. Recently, the encapsulation of the anticancer drug cisplatin in selected [Pd_2_L_4_]^4+^ cages featuring 2,6-bis(pyridine-3-ylethynyl)pyridine ligands has been studied by NMR spectroscopy, and the obtained results show that if the solvent is of sufficient polarity, metallodrug encapsulation can easily occur in the hydrophobic cavity of the cage despite the absence of specific host-guest interactions 150. Conversely, polar solvent molecules capable of forming hydrogen-bond networks (including water) are likely to prefer not to be encapsulated by hydrophobic cage cavities, and will not compete with the cisplatin molecules. This hypothesis is corroborated by previous studies on M_4_L_6_ metallacages for which guest encapsulation in polar protic solvents, such as water, appears to be driven by initial desolvation of the guest with concomitant rearrangement of the hydrogen bond networks in solution, more than by host-guest interactions alone 151.

Another strategy to consider when designing a drug delivery system is the controlled guest release of the drug at the intended target site, namely the tumor tissue. SCCs offer several options to achieve such controlled release, including competitive guest uptake 111, 152, light induced structural conformation change 153, 154, and redox mediated structural conformation changes 155, 156. Although there are currently few examples of release mechanisms for therapeutic guest molecules, it can be envisioned that the pioneering proof-of-concept studies on the reversible control over the guest encapsulation event 157, as well as guest release studies on anionic, cationic and neutral guest molecules, will also be applicable to therapeutic payloads.

Finally, an intriguing aspect of SCCs is the possibility to design supramolecular tumor-targeted modalities that combine detection and treatment through the self-assembly of emissive, metal-based coordination complexes. In fact, most ligands used to form SCCs, especially by edge-directed self-assembly, are highly conjugated systems endowed with luminescence properties 158. Moreover, additional modifications and functional groups including luminescent tags have been reported 159, 160. However, in most cases, upon cage formation and coordination of the ligands to the metal ions, the so called “heavy metal effect” dramatically induces loss of luminescence of the final SCC 117, with some exceptions 161. Modulation of the ligands and insertion of non-aromatic linkers allowed in some cases the maintenance of the luminescent properties of the attached fluorophore 159, but further studies are necessary to develop the full potential of SCCs as imaging tools in cells and tissues.

Certainly, the combination of SCCs with radioactive isotopes for diagnosis and therapy goes in this direction. Due to the huge combinatorial flexibility of SCC and SOCs towards the combination of ligands and metals, there are only few limitations of what the compounds in principle can be used for. The presented existing examples are limited to PET and MRI, but in the future also other techniques like X-ray CT might be interesting targets for SCC and SOC application. The SCC/SOCs could also combine existing tracers (like it was shown for technetates) and may enable orthogonal imaging using different techniques (e.g. guest for PET, host for MRI).

Overall, the initial examples mentioned above show that supramolecular metal-based complexes could be used to create self-formulating drugs. In fact, the self-assembly of therapeutic agents into well-defined supramolecular nanostructures is an emerging concept in both fields of molecular assembly and drug delivery to obtain one-component nanomedicines of high reproducibility.
